# Honeysuckle Berry (*Lonicera caerulea* L.) Inhibits Lipase Activity and Modulates the Gut Microbiota in High-Fat Diet-Fed Mice

**DOI:** 10.3390/molecules27154731

**Published:** 2022-07-24

**Authors:** Jong-Yeon Kim, You-Suk Lee, Eun-Jung Park, Hae-Jeung Lee

**Affiliations:** 1 Department of Food Science and Biotechnology, Gachon University, Seongnam-si 13120, Gyeonggi-do, Korea; whddus95@gachon.ac.kr; 2 Department of Food and Nutrition, Gachon University, Seongnam-si 13120, Gyeonggi-do, Korea; ysleeyun@gachon.ac.kr; 3 Institute for Aging and Clinical Nutrition Research, Gachon University, Seongnam-si 13120, Gyeonggi-do, Korea

**Keywords:** *Lonicera caerulea* L., obesity, pancreatic lipase inhibition, gut microbiota

## Abstract

Honeysuckle berry (HB, *Lonicera caerulea* L.) is an oriental herbal medicine reported to have beneficial effects on metabolic disorders, such as obesity and non-alcoholic fatty liver disease. The fruit part of HB is rich in anthocyanin, a type of polyphenol. Most studies credit the antioxidant and anti-inflammatory properties of HB as the mechanisms of its effectiveness. This study investigated the inhibitory effects of HB on lipase using an in vitro assay and the modulatory effect of HB on gut microbiota in high-fat diet (HFD)-fed mice. HB inhibited pancreatic lipase activity with IC_50_ values of approximately 0.47 mg/mL. The fecal triglyceride (TG) levels were higher from the HFD of the HB-fed mice than they were for the control mice. Moreover, the fecal microbiota from the HFD of the HB-fed mice had relatively lower *Firmicutes* and higher *Bacteroidetes* than that from the HFD-only mice. These results suggest that HB modulates gut microbiota composition, which may contribute to body fat reduction. Hence, HB could present a useful agent for treating metabolic diseases through lower TG uptake and the regulation of gut microflora.

## 1. Introduction

Obesity is caused by the overconsumption of food and insufficient energy expenditure, and the prevalence of obesity is increasing worldwide [1,2]. Obesity is closely associated with non-communicable diseases, such as cardiovascular disease, type 2 diabetes, and cancer [3,4,5,6]. Lipid accumulation caused by obesity affects fat mass and adipocyte size. The enlarged adipocyte size (hypertrophy) and increased number of adipocytes (hyperplasia) are features of obese adipose tissue [7]. Adipogenesis, lipogenesis, lipid oxidation, and thermogenesis are well-known mechanisms for lipid accumulation [8].

Lipid metabolism is essential for controlling obesity, but there is another mechanism of obesity treatment from a different perspective. One way to reduce the extra energy that is synthesized as body fat is to interfere with the digestion of fat or make it difficult for its absorption in the small intestine [9].

Orlistat (tetrahydrolipstatin, or its commercial name, Xenical) is the best-known pancreatic lipase inhibitor currently used to treat obesity [9,10]. Orlistat is derived from *Streptomyces toxitricini* and blocks the absorption of lipids to inhibit lipid accumulation as described above [11]. On the other hand, side effects, such as gastrointestinal disorders and the inhibition of absorption of fat-soluble vitamins, have recently been reported [12]. In addition, orlistat is sold as a prescription drug in Korea and other countries. The development of natural substances, such as lipase inhibitors and lipid absorption inhibitors, is required to treat and prevent obesity safely [13].

Dysbiosis causes systemic inflammation that is associated with a higher risk of obesity [14]. The gut microbiota can be classified into harmful bacteria, which are potential pathogens, and beneficial bacteria [15]. Despite the controversial opinions, most studies have found that the gut microbiota of obese humans and mice have a relatively higher level of *Firmicutes/Bacteroidetes* ratio compared to their normal-weight counterparts [16].

Honeysuckle berry (HB, *Lonicera caerulea* L.) is used as a traditional medicine because of its antioxidant and anti-inflammatory properties [17,18,19]. HB has an ameliorating effect on metabolic diseases, such as non-alcoholic fatty liver disease and type 2 diabetes [20,21]. Our previous study reported that HB has anti-sarcopenic obesity effects by reducing body weight and increasing muscle mass in high-fat diet (HFD)-fed mice [22], and we hypothesized that HB regulates the aberrant gut microbiota due to an HFD as a follow-up idea. To the best of the authors’ knowledge, there have been few studies on obesity due to lipase inhibiting activity and the modulation of the gut microbiota, even though many studies have examined the antioxidant and anti-inflammatory effects of HB. Therefore, this study examined whether HB has preventive effects on obesity by inhibiting pancreatic lipase activity and the modulation effect on the gut microbiota in HFD-fed obese-induced mice.

## 2. Results

### 2.1. Effects of HB on Pancreatic Lipase Inhibition and TG Contents in the Feces

**Figure 1:**
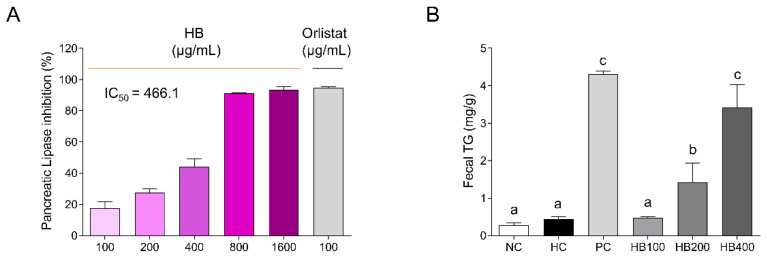
Effects of HB on pancreatic lipase inhibition and TG contents in the feces for HFD-fed mice. (**A**) Pancreatic lipase inhibition of HB; (**B**) fecal TG contents, NC–normal diet control; HC–high-fat diet (HFD) control; PC–HFD with orlistat at 20 mg/kg; HB100–HFD with HB 100 mg/kg; HB200–HFD with HB 200 mg/kg; and HB400–HFD with HB 400 mg/kg. All data are mean ± SD. Significant differences among six groups are expressed as different letters based on one-way ANOVA (*p* < 0.05), followed by Duncan’s multiple range tests.

To examine the inhibitory effect of HB on lipase activity, a pancreatic lipase inhibition assay was carried out. When the effect of HB was examined at different concentrations, HB inhibited lipase activity in a concentration-dependent manner (Figure 1A). HB, at 800 μg/mL, had the same inhibitory effect as the positive control orlistat, with an IC_50_ value for the inhibition of pancreatic lipase activity of approximately 466 μg/mL. Consistent with these findings, the fecal TG levels in HB groups increased significantly in a concentration-dependent manner. (Figure 1B). The HB group with the highest concentration produced an almost identical outcome to the PC group.

### 2.2. Effects of HB on Fat Accumulation in the Mice Model of HFD 

**Figure 2:**
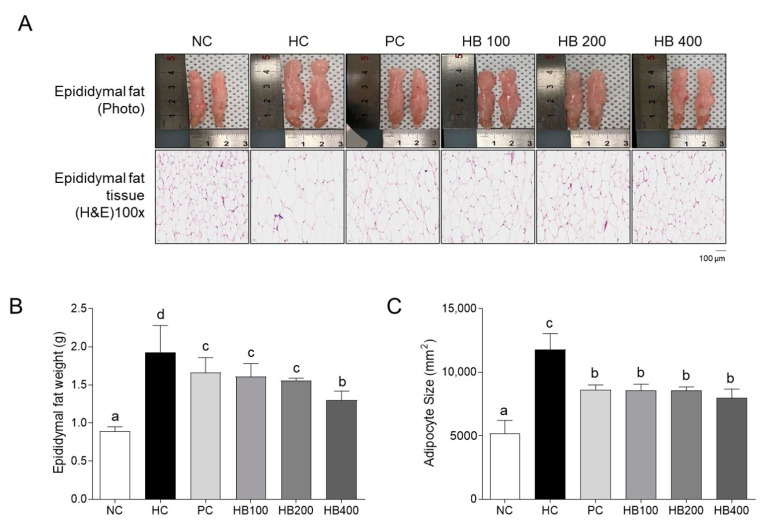
Effects of HB on the epididymal fat weight and adipocyte size in HFD-fed mice. (**A**) Photography of epididymal fat and hematoxylin and eosin (H&E) staining of epididymal fat tissue; (**B**) epididymal fat weight; and (**C**) average adipocyte cell size. All data are mean ± SD. Significant differences among six groups are expressed as different letters based on one-way ANOVA (*p* < 0.05), followed by Duncan’s multiple range tests.

To confirm the effect of HB on white adipose tissue fat accumulation, a visual examination, weight measurement, and hematoxylin and eosin (H&E) staining of epididymal fat were performed at the end of the experiment (Figure 2A). Visual inspection analysis showed that the epididymal fat size of the HC group was larger than that of the other groups (NC, PC, HB100, HB200, and HB400). In addition, the epididymal fat tissue weight and adipocyte size of the HB groups were reduced significantly compared to those of the HC group (Figure 2B,C).

### 2.3. Effects of HB on Lipid Metabolism in Adipose Tissue

**Figure 3:**
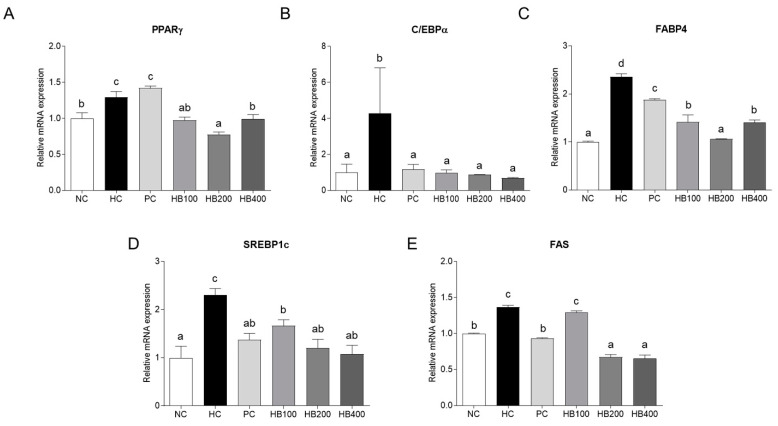
Effects of HB on the expression levels of genes related to the lipid metabolism in epididymal fat tissue of HFD-fed mice. All data are mean ± SD. Significant differences among six groups are expressed as different letters based on one-way ANOVA (*p* < 0.05), followed by Duncan’s multiple range tests.

The adipogenesis and lipogenesis regulator genes were examined to determine the effects of HB on the lipid metabolism in adipose tissue. For adipogenesis, mRNA expressions of peroxisome proliferator-activated receptor-γ (PPARγ), CCAAT/enhancer-binding protein alpha (C/EBPα), and fatty acid-binding protein 4 (FABP4) in the HC group were significantly higher than in the NC group. The PPARγ, C/EBPα, and FABP4 mRNA expression levels were significantly lower in the HB group than in the HC group (Figure 3A–C). For lipogenesis, sterol regulatory element binding protein 1c (SREBP-1c) and fatty acid synthase (FAS) mRNA levels showed similar results to adipogenesis gene levels (Figure 3D,E).

### 2.4. Effects of HB on Thermogenesis-Related Genes Expression in Adipose Tissue

**Figure 4:**
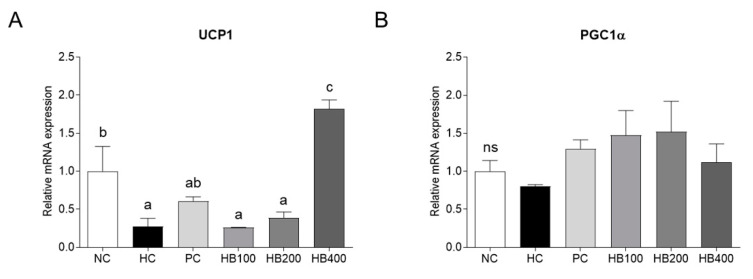
Effects of HB on the expression levels of genes related to thermogenesis in the epididymal fat tissue of HFD-fed mice. All data are mean ± SD. Significant differences among six groups are expressed as different letters based on one-way ANOVA (*p* < 0.05), followed by Duncan’s multiple range tests.

The levels of Uncoupling protein 1 (UCP-1) and PPARγ coactivator 1-alpha (PGC1α) expression were quantified by qRT-PCR to evaluate the effects of HB on thermogenesis in the adipose tissue. There was a significant up-regulation of the mRNA expression of UCP-1 in the HB400 group compared to the NC, PC, HB100, and HB200, as well as the HC group. In addition, the mRNA expression of PGC1α tends to be upregulated in the HB100, HB200, and HB400 groups compared to that in the HC group, but there was no significant difference (Figure 4A,B).

### 2.5. Effects of HB on the Composition of Fecal Microbiota

**Figure 5:**
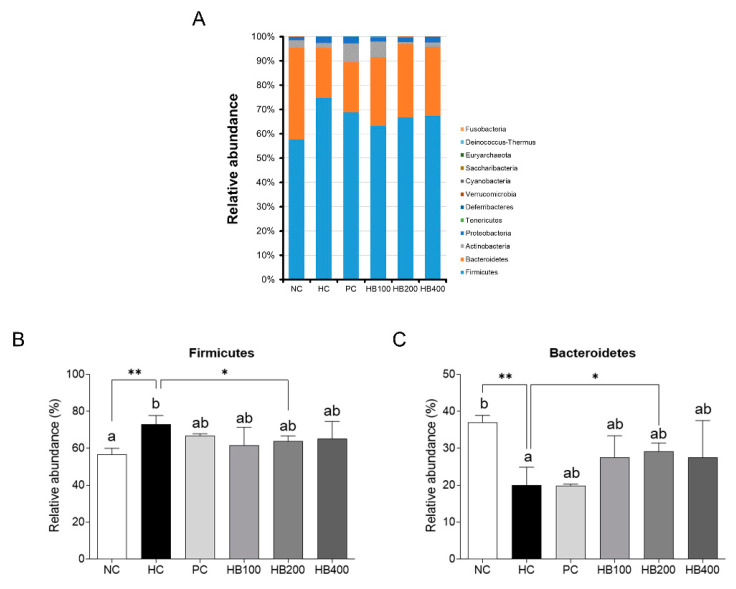
Fecal microbial relative abundance of phyla. (**A**) Bar plot represent the relative bacterial phylum abundance. Colors are used to distinguish each taxon. From the bottom, *Firmicutes*, *Bacteroidetes*, *Actinobacteria*, *Proteobacteria*, *Tenericutes*, *Deferribacteres*, *Verrucomicrobia*, *Cyanobacteria*, *Saccharibacteria*, *Euryarchaeota*, *Deinococcus-Thermus*, and *Fusobacteria* are shown. All data are mean ± SD. Significant differences among six groups are expressed as different letters based on one-way ANOVA (*p* < 0.05), followed by Duncan’s multiple range tests. * *p* < 0.05, ** *p* < 0.01 versus HC group. The group means were compared by Student’s *t*-test.

Fecal samples from each experimental group were analyzed to determine the effects of HB on the composition of fecal microbiota. In the case of the phylum level, the portion of *Firmicutes* and *Bacteroidetes* in the HC group was significantly different from the NC group and the HB200 group (Figure 5A–C). 

**Figure 6:**
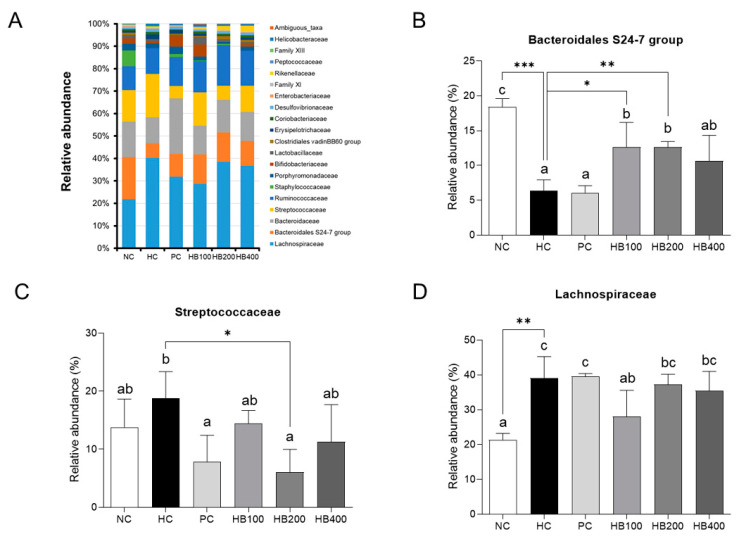
Fecal microbial relative abundance of family. (**A**) Bar plot of relative bacterial family abundance. All data are mean ± SD. Significant differences among six groups are expressed as different letters based on one-way ANOVA (*p* < 0.05), followed by Duncan’s multiple range tests. * *p* < 0.05, ** *p* < 0.01, *** *p* < 0.001 versus HC group. The group means were compared by Student’s *t*-test.

**Figure 7:**
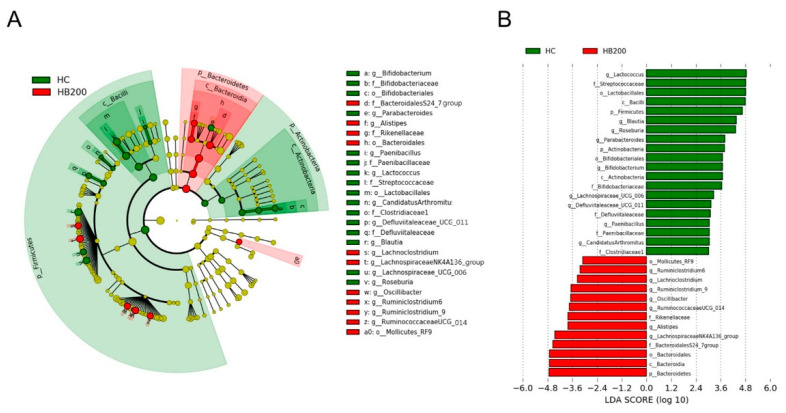
(**A**) Taxonomic cladogram and (**B**) linear discriminant analysis (LDA), obtained from LEfSe analysis of the gut microbiota in HC and HB200 groups. The only taxa meeting an LDA-significant threshold of >3 are shown.

HB also modulated the composition of the family level of the gut microbiota, increasing the *Bacteroidales S24-7*, while reducing *Streptococcaceae* and *Lachnospiaceae*, producing different results to that of the HC group (Figure 6A–D). A LEfSe tool was used to determine the dominant microbiota between the HC group and HB200 group. A comparison of the HC and HB200 groups revealed that 20 phylotypes had increased, while 13 phylotypes had decreased (Figure 7). Specifically, *Firmicutes* were found to be the major phylum of gut microbiota in the HC group, while *Bacteroidetes* were found in the HB200 group. The HB200 group increased the abundance of microbiota in the *Bacteroidia* class, *Bacteroidales* order, *BacteroidalesS24-7* group family, *Lachnospiraceae NK4A136* group genus, *Alistipes* genus, *Rikenellaceae* family, *Ruminococcaceae UCG-014* genus, *Oscillibacter* genus, *Ruminiclostridium 9* genus, *Lachnoclostridium* genus, *Ruminiclostridium 6* genus, and Mollicutes RF9 order. 

## 3. Discussion

This study confirmed the effects of HB on obesity in HFD-fed mice. The HB treatment alleviated the fat accumulation and inhibited the adipocyte hypertrophy of epididymal fat tissue. When it comes to the critical mechanisms for lipid accumulation, adipogenesis involves a cascade of transcriptional factors, including PPARγ, C/EBPα, and FABP4, which differentiate the new adipocytes from precursor cells [8,23,24]. Lipogenesis involves another transcription factor, SREBP1c, and its downstream target, FAS [25,26]. UCP1 and PGC1α are involved in thermogenesis and mitochondrial biogenesis in adipose tissue [27,28]. In this study, the expression of SREBP-1c and C/EBPα, a key regulator of lipid metabolism, changed after administering the HFD-fed mice with HB. These findings are consistent with a previous study showing that the blue honeysuckle extract (BHe) has anti-obesity effects in HFD-fed mice [29]. UCP1 acts in thermogenesis, protects against oxidative stress, and controls energy expenditure, leading to anti-obesity effects [30]. PGC1α is deacetylated by increasing SIRT1 concentration to activate the genes for fat oxidation [31,32]. The expression of UCP1 and PGC1α was upregulated after administering HB, contributing to the suppression of adipogenesis in adipocytes. These results suggest that HB regulates the mRNA expression of SREBP-1c and C/EBPα, which induces lipid metabolism and the expression of UCP1 and PGC1α, which causes thermogenesis, making it helpful for improving obesity.

Although previous research has revealed the health functional properties of HB, few studies have examined the effects of HB on lipase activity and the gut microbiota. In this study, the pancreatic lipase inhibition assay test revealed that HB inhibited pancreatic lipase activity. Pancreatic lipase is a key enzyme in the digestion of dietary fat [33]. This enzyme hydrolyzes dietary TG into fatty acids and monoglycerides, which are absorbed by the body [33]. Subsequently, fatty acids are re-synthesized to TG, which accumulates in the tissue and leads to obesity [34]. Therefore, the inhibition of pancreatic lipase suppresses fat absorption and accumulation by excreting fat out of the body [10]. Anthocyanin, a type of polyphenol, is a flavonoid found mainly in fruits and flowers, which has been shown to inhibit pancreatic lipase activity [35,36]. When the phytochemical composition of the extract used in this study was examined, it was discovered that the presence of anthocyanins, chlorogenic acid, rutin, and ellagic acid was observed (Figures S1 and S2, Tables S1 and S2). As expected, cyanidin 3-O-glucoside (C3G), a type of anthocyanin, was a major component, suggesting that it inhibits pancreatic lipase activity. Furthermore, we found HB increased the fecal TG level in this study and confirmed that HB decreased the serum TG level in our previous study [22]. These results support our results, suggesting that HB could be developed as a lipid absorption inhibitor.

The role of the gut microbiota in homeostasis, metabolism, adiposity, and energy regulation, as well as in vitamin production and nutrient harvesting, which together are related to obesity and obesity-associated disorders, continues to be investigated [37,38]. Anthocyanins showed prebiotic properties that are catabolized by the gut microbiota [39] and have been found to play a role in alleviating obesity by decreasing the *Firmicutes/Bacteroidetes* ratio [40,41]. This study confirmed that the HFD-fed mice increased the number of *Firmicutes* and decreased the number of *Bacteroidetes*. On the other hand, mice fed with the HFD with HB decreased in their number of *Firmicutes* and increased in *Bacteroidetes*. Furthermore, anthocyanin supplementation reversed the decrease of short-chain fatty acids (SCFAs) caused by the HFD [42]. Also, the *Lachnospiraceae NK4A136* group and *RuminococcaceaeUCG-014* group, which produce butyrate, were found to be enriched in the HFD-fed mice treated with an anti-obesity agent [43,44]. In this study, HB extract increased these two floras. Our findings are consistent with previous studies [43,44], suggesting that HB extract regulates fecal microbiota via their prebiotic properties and has a beneficial effect on HFD-induced obesity in mice.

## 4. Materials and Methods

### 4.1. Preparation of HB Extract

Raw HB fruits were extracted as described previously [22]. The amount of C3G, one of the major anthocyanins in HB extract, was measured to be 11.12 mg/g [22]. The HB extract was stored at −20 °C and dissolved in a 0.5% aqueous solution of carboxymethyl cellulose sodium (CMC-Na, TCI, Tokyo, Japan).

### 4.2. Pancreatic Lipase Inhibition Assay

The substrate p-nitrophenyl butyrate (p-NPB) was used to perform the pancreatic lipase inhibition assay and the method was slightly modified from the previous assay method [33]. Briefly, either the various concentrations of HB or 100 μg/mL Orlistat were mixed with the Tris buffer and enzyme buffer. After 15 min at 37 °C, the substrate solution was added and incubated for 30 min at 37 °C. All the materials used in the assay were purchased from Sigma–Aldrich (St. Louis, MO, USA). The pancreatic lipase inhibitory activity was measured at 405 nm. The inhibitory activity (%) was calculated as follows:

Lipase inhibition (%) = [1 − (B − b)/(A − a)] × 100

where A is the activity without the sample with the enzyme, and a denotes the activity without the sample and enzyme. B is the activity of the sample with the enzyme and b is the activity of the sample without the enzyme.

### 4.3. Animals and Treatment

Forty-eight six-week-old male C57BL/6 mice were purchased from Orient Bio Co. Ltd. (Seongnam-si, Gyeonggi-do, Korea) and maintained in a temperature and humidity-controlled room on a 12 h/12 h light/dark cycle at Gachon University, Korea. They were given access to food and drinking water ad libitum over a one-week acclimation period. The mice were randomly split into six groups of eight mice each: NC (normal diet control, 10% calories from fat, D12450B, Research Diets, New Brunswick, NJ, USA), HC (HFD, 45% calories from fat; D12451; Research Diets, NJ, USA), PC (positive control, HFD with orlistat 20 mg, TCI, Tokyo, Japan), HB100 (HFD with HB100 mg/kg), HB200 (HFD with HB200 mg/kg), and HB400 (HFD with HB400 mg/kg). The experimental extracts were dissolved in a 0.5% aqueous solution of CMC-Na (TCI, Tokyo, Japan). Orlistat and different doses of HB were administered orally to PC and the experimental group mice, respectively, whereas the solvent was administered orally to the NC and HC group mice. For eight weeks, oral administration was performed once per day. All the animals were cared for, operated in compliance with Gachon University’s guidelines for the care and use of laboratory animals (reference number: GIACUC-R2020009).

### 4.4. Epididymal Fat Tissue and Feces Collection

After 12 h of fasting, the mice were euthanized with CO_2_. The epididymal fat tissue was dissected and weighed immediately. The feces of the mice were collected the day before the end of the experiment. All samples were stored at −80 °C until analysis.

### 4.5. Fecal Triglyceride Analysis

After drying for 24 h at 60 °C in a drying oven (DAIHAN Scientific Co., Ltd. Wonju, Korea), 250 mg of fecal samples were homogenized with 2 mL of phosphate-buffered saline. Fecal fat was extracted using the method reported by Folch et al. [45]. The fecal triglyceride (TG) levels were determined using a TG enzymatic assay kit (Asanpharm, Hwaeong, Korea).

### 4.6. Histological Analysis

H&E staining was performed according to the previously described method [22]. Briefly, the epididymal fat tissue was fixed, waxed, cut, and stained with H&E. Subsequently, sample images were taken using a Nikon DS-Ri2 camera (Nikon, Tokyo, Japan).

### 4.7. Quantitative Reverse-Transcription Polymerase Chain Reaction (qRT-PCR)

The total RNA was extracted from the epididymal fat tissue, and the cDNA was synthesized from 50 ng/μL RNA. qRT-PCR was performed using an ABI QuantStudio 3 (Applied Biosystems, Foster City, CA, USA). Table 1 lists the primer sequences used for qRT-PCR. 

### 4.8. Microbiota Analysis

Fecal microbiota analysis was performed pursuant to a previous study with some modifications [46]. Briefly, the QIAamp PowerFecal DNA Kit (QIAGEN, Hilden, Germany) was used to extract the genomic DNA from feces samples. The bacterial 16S rRNA V4 region was amplified with unique 8 bp barcodes and sequenced on the Illumina iSeq 100 system according to standard protocol [47]. The sequences were processed by the Quantitative Insights into Microbial Ecology (QIIME) pipeline [48] using the SILVA 128 database [49]. Linear discriminate analysis (LDA) effect size (LEfSe, http://huttenhower.sph.harvard.edu/galaxy/, accessed on 11 May 2022) was performed to compare the taxon microbes between HC and HB200 groups.

### 4.9. Statistical Analysis

Statistical differences of all data were analyzed using Duncan’s multiple range test after one-way analysis of the variance. Student’s *t*-test, using SPSS 25 (SPSS Inc., Chicago, IL, USA), was performed for two group comparisons. *p* values < 0.05 were considered significant.

## 5. Conclusions

Overall, this study showed that HB significantly inhibits pancreatic lipase activity, reducing dietary fat absorption. The anti-obesity efficacy of HB was comparable to that of the positive control, orlistat. Moreover, HB positively modulates the gut microbiome. These findings provide scientific evidence that HB prevents obesity and modulates the gut microbiota in HFD-induced obese mice.

## Figures and Tables

**Table 1:** Primer sequence used for qRT-PCR.

Genes	Forward Primer (5′-3′)	Reverse Primer (5′-3′)	Accession Number
*Pparγ*	TTTTCAAGGGTGCCAGTTT	AATCCTTGGCCCTCTGAGAT	NM_001127330.2
*C/ebp* *α*	TTACAACAGGCCAGGTTTCC	GGCTGGCGACATACAGTACA	NM_001287523
*Fabp4*	TCACCTGGAAGACAGCTCCT	AATCCCCATTTACGCTGATG	NM_024406.3
*Srebp1c*	ATCGCAAACAAGCTGACCTG	AGATCCAGGTTTGAGGTGGG	NM_011480
*Fas*	TTGCTGGCACTACAGAATGC	AACAGCCTCAGAGCGACAAT	NM_007988.3
*Ucp1*	CTTTGCCTCACTCAGGATTGG	ACTGCCACACCTCCAGTCATT	NM_009463.3
*Ppargc1α*	ATGTGTCGCCTTCTTGCTCT	ATCTACTGCCTGGGGACCTT	NM_008904.2
*Actb*	CCACAGCTGAGAGGAAATC	AAGGAAGGCTGGAAAAGAGC	NM_007393.5

qRT-PCR, quantitative reverse transcription-polymerase chain reaction; *Pparγ*, Peroxisome proliferator-activated receptor-gamma; *C/ebpα*, CCAAT/enhancer-binding protein alpha; *Fabp4*, Fatty acid-binding protein 4; *Srebp1c*, Sterol regulatory element binding protein 1c; *Fas*, Fatty acid synthase; *UCP*, Uncoupling protein; *Ppargc1a*, Peroxisome proliferator-activated receptor-gamma coactivator.

## Data Availability

The data presented in this study are available in supplementary material.

## Supplementary Materials

The following supporting information can be downloaded at: https://www.mdpi.com/article/10.3390/molecules27154731/s1, Figure S1: high-performance liquid chromatography (HPLC) of (A) phenolic compounds standards and (B) HB extract; Figure S2: high-performance liquid chromatography (HPLC) of (A) anthocyanin standard and (B) HB extract; Table S1: The identified phenolic compounds except anthocyanin in HB extracts by high performance liquid chromatography; Table S2: The identified anthocyanin in HB extracts by high performance liquid chromatography.

## Sample Availability

Samples of the compounds are not available from the authors.
