# Supplementary material for: Honeysuckle Berry (Lonicera caerulea L.) Inhibits Lipase Activity and Modulates the Gut Microbiota in High-Fat Diet-Fed Mice
Source: Molecules. 2022 Jul 24;27(15):4731. doi: 10.3390/molecules27154731 (PMC9330072; doi:10.3390/molecules27154731)
Supplement: Supplementary file 1 [file molecules-27-04731-s001.zip › molecules-1796067-supplementary.pdf]

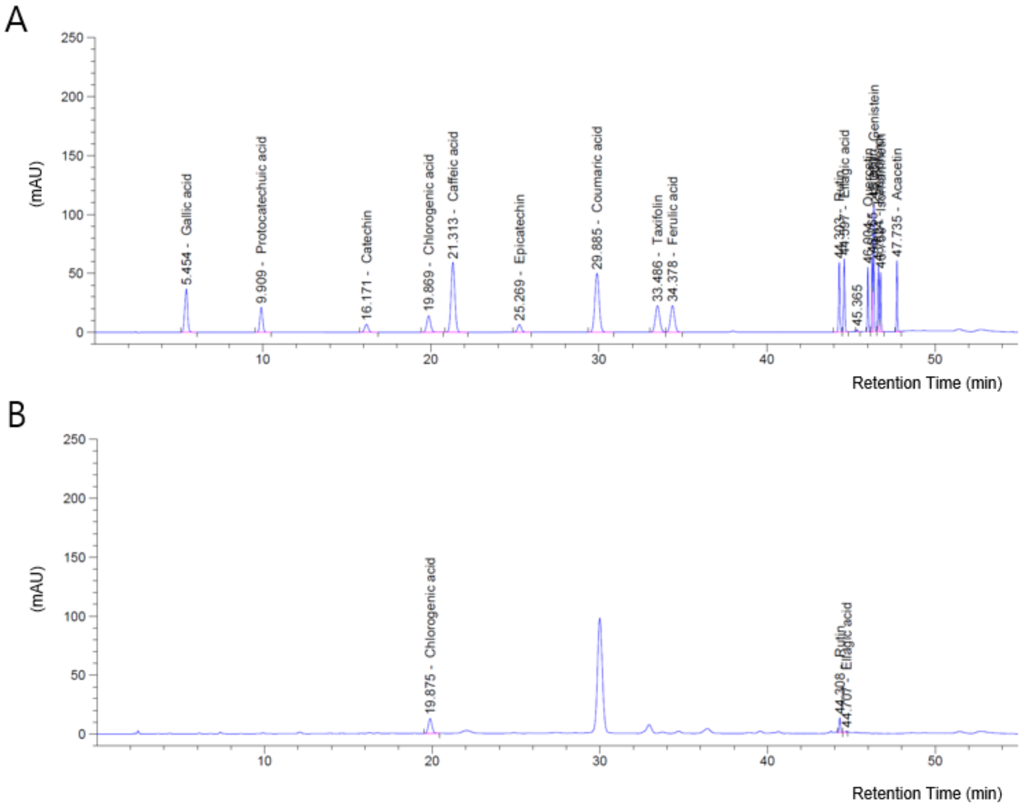

Fig. S1. High-performance liquid chromatography (HPLC) of (A) phenolic compounds standards and (B) HB extract.

Table S1. The identified phenolic compounds except anthocyanin in HB extracts by High Performance Liquid Chromatography

Retention Time (min)	Compound	Molecular weight	Structural formula
19.875	Chlorogenic acid	354.3	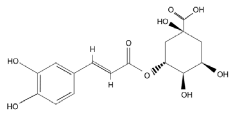
44.308	Rutin	610.5	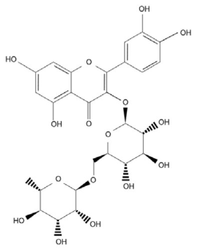
44.707	Ellagic acid	302.2	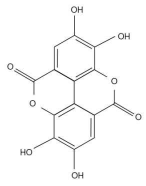

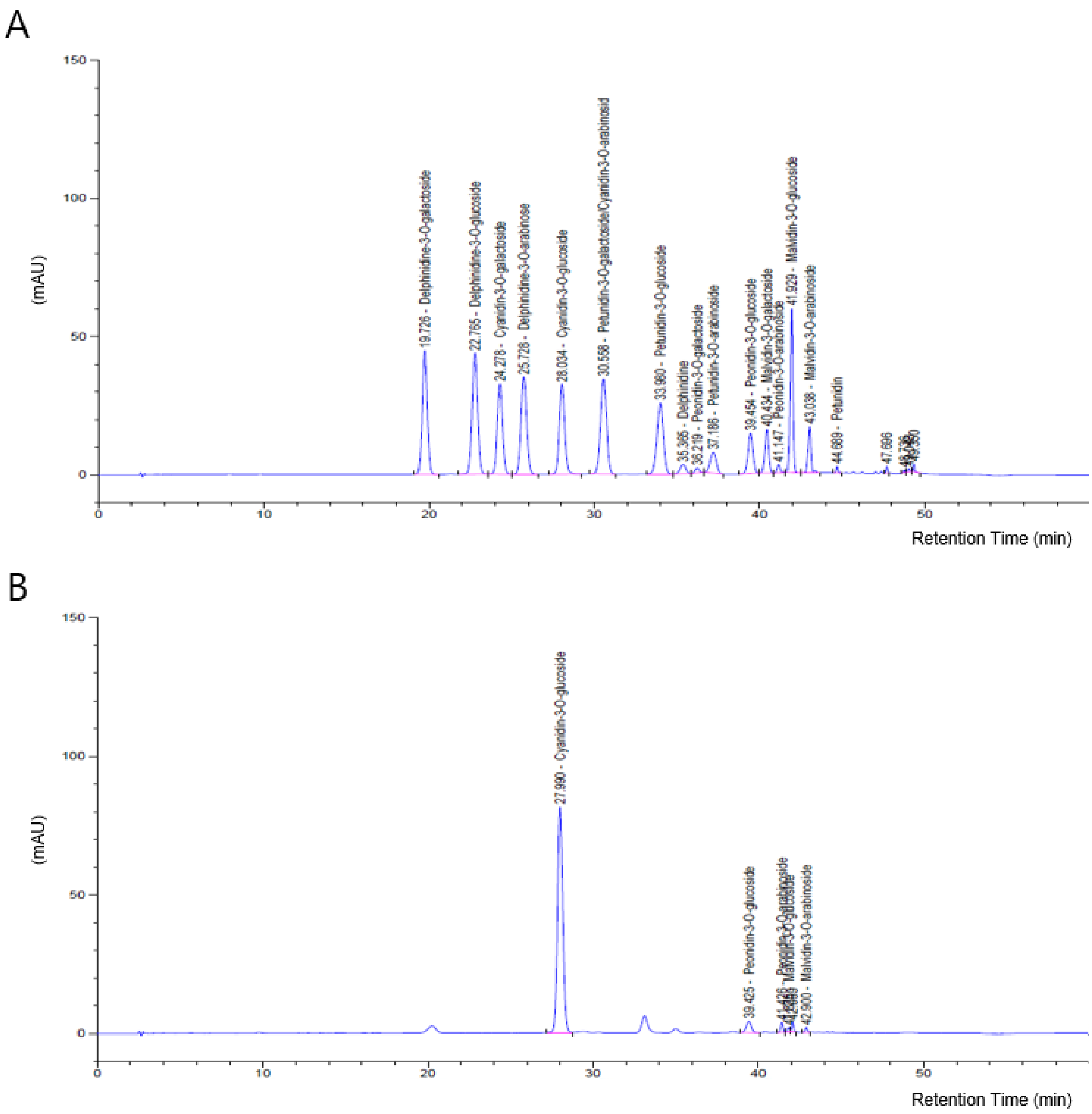

Fig. S2. High-performance liquid chromatography (HPLC) of (A) anthocyanin standard and (B) HB extract.

Table S2. The identified anthocyanin in HB extracts by High Performance Liquid Chromatography

Retention Time (min)	Compound	Molecular weight	Structural formula
27.990	Cyanidin-3-O-glucoside	484.8	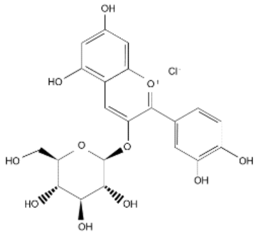

39.425	Peonidin-3-O-glucoside	463.4	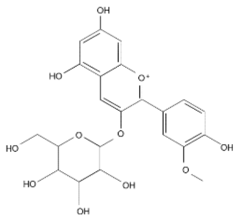
41.426	Peonidin-3-O-arabinoside	433.4	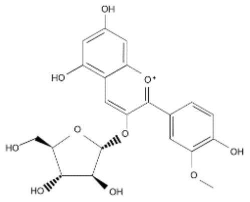
41.825	Malvidin-3-O-glucoside	528.9	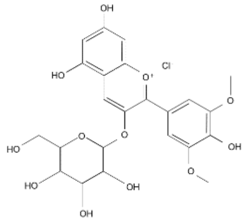
42.900	Malvidin-3-O-arabinoside	498.9	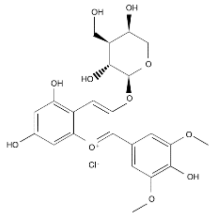

Analyses of Phenolic compounds in HB extract

Phenolic compounds of the HB extract were analyzed using the High-Performance Liquid Chromatography (HPLC) system (1260 Infinity II, Agilent, Santa Clara, CA, USA).

For the phenolic compounds except anthocyanin, Agilent Eclipse XDB C18 (5um, 4.6 I.D. × 250mm) column was used. Mobile phases A (water with 0.5% Phosphoric acid) and B (Methanol) were conducted with gradients (0–40 min, 92:8; 40–45 min, 66:34; 45–48 min, 2:98; 48–50 min, 2:98; 50–55 min, 92:8, v/v). The solvent flow rate was set at 1.0 mL/min. The HPLC-UVD system was operated at a gain of 1 and drift column oven temperature of 40 °C. The runs were monitored for phenolic components without anthocyanin at 280 nm. The 0.3g of samples were dissolved in 80% methanol followed by membrane (0.45 μm). Solvents for HPLC experiments were purchased from Burdick &Jackson (Morristown, NJ, USA). Standard materials were commercially purchased (Gallic acid, Catechin, Chlorogenic acid, Caffeic acid, Epicatechin, Coumaric acid, Taxifolin, trans-Ferulic acid, Rutin, Ellagic acid, Quercetin, Kaempferol, Isorhamnetin, and Acacetin were purchased from Sigma–Aldrich, St. Louis, MO, USA; Protocatechuic acid was purchased from HWI; Luteolin was purchased from PhytoLab, Genistein was purchased from Wako).

For the anthocyanin, Shiseido UG 120 C18 (5um, 4.6 I.D. × 250mm) column was used, and analyzed according to the USP 37 NF32 (Powdered Bilberry Extract, Content of

Anthocyanosides and Anthocyanidins). Briefly, mobile phases A (water with 10% Formic acid acid) and B (Methanol : Fromic acid : D.W. = 45 : 45 : 20 : 80) were conducted with gradients (0–35 min, 93:7; 35–45 min, 75:25; 45–46 min, 35:65; 46–50 min, 0:100; 50–51 min, 0:100; 51–60 min, 93:7, v/v). The solvent flow rate was set at 1.0 mL/min. The HPLC-UVD system was operated at a gain of 1 and drift column oven temperature of 30 °C. The runs were monitored for anthocyanin at 535 nm. Standard materials (Powdered Billberry extract, USP Certificate) was purchased from Sigma–Aldrich (St. Louis, MO, USA)
